# Steric Effects Induce Geometric Remodeling of Actin Bundles in Filopodia

**DOI:** 10.1016/j.bpj.2016.03.013

**Published:** 2016-05-10

**Authors:** Ulrich Dobramysl, Garegin A. Papoian, Radek Erban

**Affiliations:** 1 Wellcome Trust/Cancer Research UK Gurdon Institute, University of Cambridge, Cambridge, United Kingdom; 2 Department of Chemistry & Biochemistry, University of Maryland, College Park, Maryland; 3 Mathematical Institute, University of Oxford, Radcliffe Observatory Quarter, Oxford, United Kingdom

## Abstract

Filopodia are ubiquitous fingerlike protrusions, spawned by many eukaryotic cells, to probe and interact with their environments. Polymerization dynamics of actin filaments, comprising the structural core of filopodia, largely determine their instantaneous lengths and overall lifetimes. The polymerization reactions at the filopodial tip require transport of G-actin, which enter the filopodial tube from the filopodial base and diffuse toward the filament barbed ends near the tip. Actin filaments are mechanically coupled into a tight bundle by cross-linker proteins. Interestingly, many of these proteins are relatively short, restricting the free diffusion of cytosolic G-actin throughout the bundle and, in particular, its penetration into the bundle core. To investigate the effect of steric restrictions on G-actin diffusion by the porous structure of filopodial actin filament bundle, we used a particle-based stochastic simulation approach. We discovered that excluded volume interactions result in partial and then full collapse of central filaments in the bundle, leading to a hollowed-out structure. The latter may further collapse radially due to the activity of cross-linking proteins, hence producing conical-shaped filament bundles. Interestingly, electron microscopy experiments on mature filopodia indeed frequently reveal actin bundles that are narrow at the tip and wider at the base. Overall, our work demonstrates that excluded volume effects in the context of reaction-diffusion processes in porous networks may lead to unexpected geometric growth patterns and complicated, history-dependent dynamics of intermediate metastable configurations.

## Introduction

Many eukaryotic cells project dynamic fingerlike protrusions, called filopodia, that are composed of a bundle of actin filaments enveloped by the cellular membrane (1, 2). Filopodia play diverse roles across many cell types. In particular, signaling via receptors on filopodial tips allows cells to sense their environment and guide chemotaxis (3). Neurons use filopodia in axonal growth cones, to determine the direction of elongation and branching (4), as well as in dendritic spine formation (5). During wound healing, knitting of filopodia protruding from epithelial cells plays an important role (6). Filopodia are also implicated in cancer progression and metastasis because of their involvement with cell motility (7). They also arise in some viral infections, creating physical connections among the hosts’ cells (8).

G-actin, an abundant and highly conserved protein, self-assembles into double helical filaments called F-actin. The latter is the fundamental building block of eukaryotic cellular cytoskeletons. F-actin structure is polarized, with polymerization at the barbed-end being more efficient by an order of magnitude compared to the pointed-end, while having similar depolymerization rates at both ends. This asymmetry, based on the hydrolysis of ATP into ADP by actin molecules, leads to treadmilling, whereby filaments can convert chemical energy stored in ATP into mechanical work of pushing against the external resistance. Hence, actin filaments are dynamic, dissipative structures that allow for fast morphological transitions in the cellular cytoskeleton in response to external and internal biochemical and mechanical cues, mediated by a vast array of signaling and regulatory proteins.

**Figure 1:**
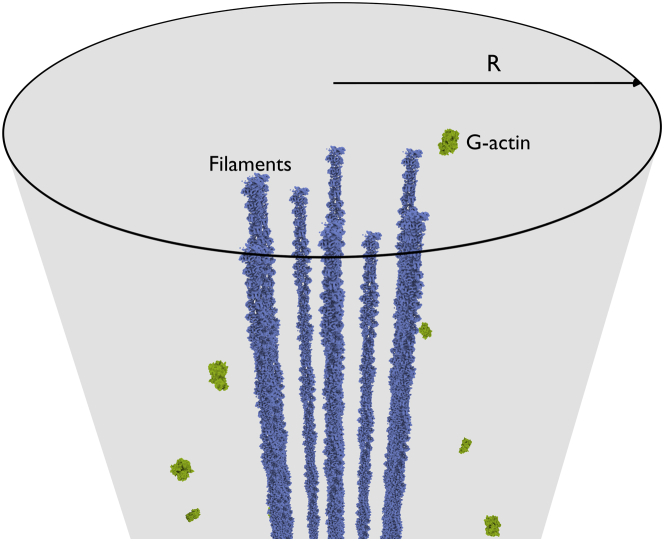
Rendering of an actin filament bundle inside a filopodium. The filaments are shown in the middle, with diffusing G-actin monomers displayed around them. The gray region indicates the volume enclosed by the membrane in our model. The filament height and G-actin position data stem from an example simulation run. Filament and G-actin structure data were taken from the PDBe database (63, 64) To see this figure in color, go online.

In filopodia, which are roughly cylindrical tubes with radius *R* ∼ 20–250 nm (9, 10), between 10 and 30 actin filaments are organized into parallel, tightly cross-linked structures (see Fig. 1). The filopodial lengths, *L*, vary considerably between cell types, ranging from a few up to several tens of microns (11, 12). Many proteins are involved in the complex machinery responsible for the formation and subsequent biological function of filopodial protrusions, including formins (13), ENA/VASP (14), and capping proteins (15), among others; however, how these proteins regulate the growth-retraction dynamics of filopodia is still not fully understood (1, 16, 17, 18).

In a series of works, Lan and Papoian (19) and Zhuravlev and Papoian (20) have proposed a theory for length regulation in filopodia. For a stationary filopodium, the key idea is to match three actin fluxes: the consumption of G-actin at the filopodial tip (*J*_*p*_) must equal the transport or diffusional flux of G-actin to the tip (*J*_*d*_) and the flux of actin subunits leaving the filopodial tube (*J*_*r*_) due to retrograde flow. The latter process is mechanically mediated by the polymerization of actin against the resistance of the filopodial membrane and, to a larger extent, by contractile dynamics of actin network inside the cell body, to which the roots of filopodial actin bundles are anchored (18, 21, 22, 23, 24, 25). In the mean-field limit, the equations, *J*_*p*_ = *J*_*d*_ = *J*_*r*_ can be solved to yield a closed form expression for the stationary length of filopodia (19). Prior models also investigated filopodial growth dynamics, using somewhat different assumptions and computational approaches (9, 11, 26). Detailed stochastic models of filopodial growth that included important regulatory proteins such as capping proteins, resulted in macroscopic length fluctuations of filopodial protrusions reminiscent of ubiquitously observed filopodial growth-retraction cycles (20). These near-critical length fluctuations arise from the amplification of molecular noise due to binding and unbinding of capping proteins to filament ends (20). In addition, Zhuravlev and Papoian (20) found that capping proteins induce bundle thinning, leading to the eventual full collapse of filopodia, suggesting a microscopic mechanism for filopodial aging and limits on their lifetime.

Excluded volume effects have been studied in the context of macromolecular crowding in cells. It has been shown that crowding introduces a size-dependent viscosity inside the cytoplasm (27), rendering the motion of proteins highly nontrivial. The complex structure of chromatin inside cell nuclei leads to a significant reduction of the effective diffusion coefficient of fluorescent probes (28), thereby possibly influencing and regulating the transcription of DNA into mRNA and the subsequent translation into proteins. Simulation studies and analytical work considering volume exclusion done on chromatin structure extracted from real nuclei via x-ray tomography showed that there exists an optimal amount of volume exclusion that minimizes the mean time for DNA-binding proteins to find their binding sites (29). Theoretical works have shown that Brownian motion with hard-sphere interactions can be captured via an effective, collective diffusion constant (30). Considerably less work has been done to study the feedback between growing cytoskeletal structures and the resulting restriction of the free diffusion of their building blocks.

Here, we show that excluded volume effects also result in filament collapse and bundle thinning. However, in contrast to the effect of capping protein, steric constraints on G-actin diffusion in the bundle interior result in geometrically preferential growth-retraction patterns. Instead of any filament in a bundle being susceptible to collapse due to capping, core filaments in the bundle are starved of G-actin and eventually collapse. G-actin monomers are proteins with an approximately ellipsoidal shape, with the semiprincipal axes of 6.7 × 4 × 3.7 nm (31). In addition, both cytosolic and filamentous actin molecules are negatively charged, resulting in their mutual electrostatic repulsion. Because a typical electrostatic screening length is on the order of 1 nm under physiological conditions (32), the electrostatic repulsion is expected to add ∼2 nm to the steric exclusion zone (33) when considering penetration of G-actin molecules in-between two F-actin filaments. In our calculations, we consider G-actin as spherical particles with a radius of 3.5 nm, averaging over its ellipsoidal semiaxes and also taking into account the screened electrostatic repulsion between G-actin and F-actin. The diameter of actin filaments is ∼7 nm (34, 35, 36). The average interfilament spacing inside a bundle of actin filaments is 12 nm (37, 38, 39) with a range of ∼10–13 nm (39). In terms of lateral geometrical placements, actin filaments in a filopodial bundle were found to be arranged on an ordered hexagonal lattice (37). Therefore, the geometry of the porous networks found in these tightly cross-linked bundles is likely to impede monomeric G-actin passing through the space between two neighboring filaments. The resulting hindered diffusion of G-actin restricts availability of G-actin in the bundle interior.

Our simulations show that the above outlined steric exclusion has profound effects on the shape of the filopodial tip. The initial transient growth phase, during which some interior filaments collapse, results in a stationary filament length configuration that is only metastable. Over time, interior filament height fluctuations drive them below a mean-field cutoff height (discussed below), when they subsequently collapse to a new metastable configuration at an intermediate height and eventually collapse completely. The partial or full collapse of an interior filament creates a new diffusion channel for G-actin to explore. These channels determine the subsequent critical lengths for the fluctuations of the remaining interior filaments. Hence, the further evolution of the inner filament heights is highly dependent on the time ordering of previous filament collapses, leading to history-dependent metastable filopodial states. Over time, these processes completely hollow out the interior of actin bundle at the filopodial top, while leaving the peripheral filaments largely intact. In terms of long-term evolution, such a hollow structure may not be stable with respect to the activity of cross-linking and motor proteins, leading to a global geometric reshaping of the filopodial actin bundle, which, in turn, would significantly diminish its mechanical stability, as discussed below.

In the next section, we discuss our methodology and tools. Following that, we present our simulation results and discuss the most salient metastable states arising from the volume exclusion effects. Subsequently, we introduce a mean-field model that allows us to gain further insight into the stability conditions of the observed states, finding that the mean-field predictions for the heights of partially collapsed filaments closely agree with the Brownian dynamics simulation results. Finally, we analyze the mechanical implications of the morphological transitions induced by the above-mentioned excluded volume effects.

## Materials and Methods

To investigate how steric interactions among filaments and G-actin monomers affect actin polymerization dynamics, we have developed a simulation model that incorporates excluded volume effects. In our model, we allow the elongation of actin filaments via binding of G-actin molecules. G-actin particles are allowed to move according to Brownian dynamics (BD, see below) and can bind to microfilaments when they enter the vicinity of a binding site on top of a filament, thereby elongating the filament by a length *δ*. Note that the real mechanism of actin polymerization at the filopodial tip may be more complicated and involves additional protein complexes such as ENA/VASP and formins. Therefore, our model should be treated as effectively averaging over these details with an effective (or renormalized) polymerization rate. The simulation model proceeds via discrete time steps Δ*t* = 100 ns, in which molecules first move via the BD step, subsequently molecules near binding sites are allowed to bind, and finally G-actin can depolymerize from the tips of filaments, freeing a molecule and reducing the filament length by *δ*. The parameter values used in this study are summarized in Table 1. We ran 200 distinct realizations of this model for 2000 s each to extract filament height trajectories over time for three different values of the interfilament spacings (taking ∼50,000 CPU h).

### Particle-based stochastic model for diffusion

Our spatially extended stochastic model for filopodial growth is confined to a spatial domain with cylindrical shape of variable length $L\left(t\right)=L_{F}\left(t\right)+25\;\text{nm}$ (depending on the length of the filopodium at a time *t*, which is given by the highest filament at $L_{F}\left(t\right)$) and radius *R* = 75 nm. G-actin monomers can enter and exit the domain via the boundary at the bottom, which is held at a constant concentration. These particles undergo BD with a fixed time step Δ*t*, i.e., their position is updated according to $X_{j}\left(t+\Delta t\right)=X_{j}\left(t\right)+\sqrt{2D\Delta t}\;\xi_{j}$ with $j=1,2,\ldots ,N_{A}\left(t\right)$, where $N_{A}\left(t\right)$ is the number of G-actin molecules at a given time *t*, and $\xi_{j}$ is a vector of independent normally distributed random numbers with zero mean and unit variance. Volume exclusion of filaments and boundaries are handled via reflection along the surface normal.

### Volume exclusion

Filaments are modeled as rigid cylinders with a finite radius $r_{F}$ placed in a hexagonal arrangement (see Lateral Position of Filaments and Initial State). To account for the spatial extent of G-actin molecules (which is assumed spherical, with the same radius $r_{F}$ ), the effective radius of filaments is given as $2r_{F}$ (and diffusing molecules are implemented as points). To facilitate this study, G-actin molecules do not mutually interact, hence we do not take queuing and crowding effects into account as this would become computationally prohibitive. The concentration of G-actin is relatively small, hence we do not expect large effects from neglected interactions of G-actin molecules.

### Molecule binding

To model the binding of molecules to filament tips, we implement a reversible binding scheme (40), with a binding radius of $\varrho =2r_{F}=7\;\text{nm}$. In this scheme, molecules become binding candidates as soon as they enter a sphere of radius ϱ around the tip of a filament. They are then allowed to bind to the site with a probability $P_{\lambda }$ per time step that they spend in the binding region (40, 41). In the vicinity of the top of the filopodium, the binding probability is modified to take the force enacted on the bundle via the membrane into account (see below). We calculate the remaining parameter $P_{\lambda }$ (binding probability) before the start of simulations using the approach described in Lipková et al. (Section 5 in (40)). To implement depolymerization, a molecule is allowed to dissociate from a filament tip with a probability $1-\text{exp}\left(-k^{-}\;\Delta t\right)$ and placed at the former position of the tip. Here, we neglect possible changes in the depolymerization rate due to the hydrolysis of actin molecules as the resulting large fluctuations in filament length occur only at actin concentrations much smaller than exist in our simulations (42).

### Membrane force

Experiments have shown that the actin polymerization rate highly depends on the force enacted on filaments by the membrane (43). This effect can be thought of as temporary envelopment of a filament tip by the membrane, sterically disallowing the binding of G-actin to the tip, thereby synchronizing the movement of the filament tips close to the membrane (19). Thus, the bare bulk polymerization rate *k*^+^ needs to be multiplied by the probability for the creation of enough space between a filament tip and the membrane to accommodate the molecule. This Brownian ratchet model allows us to take into account membrane effects without explicitly simulating the movement of the membrane by modifying the binding probability of G-actin molecules inside the binding radius of the *i*th filament (20, 44)

(1) $$P_{\lambda }^{\left(i\right)}=P_{\lambda }\text{exp}\left(-\frac{f_{i}\delta }{k_{\text{B}}T}\right),\;i=1,2,\ldots ,N,$$

where $f_{i}$ is the force on filament *i*. On the micrometer scale, the cell membrane is known to equilibrate on the order of microseconds (45), hence much faster than the average time between polymerization reactions. Therefore, $f_{i}$ is proportional to the probability of the filament tip being covered by the membrane. Assuming Gaussian-distributed membrane height fluctuations, we can follow Lan and Papoian (19) to calculate the probability of the membrane being below the filament tip

$$p_{i}=\frac{1}{2}\text{erfc}\left(\frac{\text{max}\left\{h_{j}\right\}-h_{i}}{\sigma }\right),$$

where erfc(x)=1−erf(x) is the complementary error function and $h_{j}$ is the height of the *j*th filament. The total membrane force *f* is distributed across all the filaments according to this probability, hence the force on filament *i* is $f_{i}=\left(p_{i}f/\sum_{j=1}^{N}p_{j}\right)$, which we can substitute into Eq. 1 to get the effective polymerization probability.

Wang and Carlsson (46) model the motion of a Brownian obstacle (the membrane in our case) pushed by polymerizing filaments. They disallow polymerization when the distance to the membrane is less than the monomer size and let polymerization proceed with the bare rate *k*^+^ otherwise. Das et al. (47) consider a discrete lattice model for filament polymerization against an external load that reduces the polymerization rate of the leading filament (in contact with the obstacle) only. Our model differs from both of these in that we assume a nonrigid, highly dynamic membrane as the obstacle, which can affect the polymerization of filaments even a distance below the mean membrane position (19).

### Filament polymerization and collapse

The dynamics of actin filaments are governed by the inherent asymmetry between the two ends of a filament. The barbed end (the filament tip in our simulations) exhibits a much higher polymerization rate, which leads to a treadmilling process. Actin microfilaments grow via the binding of G-actin monomers, which enhances the filament length by *δ* = 2.7 nm. The binding algorithm implemented in our simulations is discussed above. The bulk polymerization rate is given by $k^{+}=11.6\;\mu {\text{M}}^{-1}\;{\text{s}}^{-1}$ from which we calculate the binding probability for a given binding radius. The binding radius cannot be larger than half of the distance between filaments such that the binding region does not overlap with excluded volume regions, thereby artificially lowering the effective binding rate. During a depolymerization reaction, the filament’s height is reduced by a length *δ* and a new G-actin molecule is placed at the former position of the filament tip. Enhanced depolymerization processes at the spiked ends of actin filaments in the lamellipodium lead to a pulling-back motion of filaments called “retrograde flow”. We incorporate this in our simulation model via a constant effective velocity of filament tips *v*. During each time step, each filament’s height shrinks by *v*Δ*t*, in addition to any depolymerization reaction occurring during this time step.

### Lateral position of filaments and initial state

Filaments are placed in a hexagonal arrangement with an initial height of 100 nm. The in-plane coordinates ${\vec{x}}_{i}$ of the *N* = 19 filaments are given by

$$\begin{array}{l} \left(0,0\right),\;\left(0,d\right),\;\left(\sqrt{3}d/2,d/2\right),\;\left(\sqrt{3}d/2,-d/2\right),\\ \left(0,-d\right),\;\left(-\sqrt{3}d/2,-d/2\right),\;\left(-\sqrt{3}d/2,d/2\right),\\ \left(\sqrt{3}d/2,3d/2\right),\;\left(\sqrt{3}d,0\right),\;\left(\sqrt{3}d/2,-3d/2\right),\\ \left(-\sqrt{3}d/2,-3d/2\right),\;\left(-\sqrt{3}d,0\right),\;\left(-\sqrt{3}d/2,3d/2\right),\\ \left(0,2d\right),\;\left(\sqrt{3}d,d\right),\;\left(\sqrt{3}d,-d\right),\;\left(0,-2d\right),\\ \left(-\sqrt{3}d,-d\right),\;\left(-\sqrt{3}d,d\right), \end{array}$$

with *d* as the next-neighbor distance. The hexagonal arrangement of microfilaments in a bundle has been observed in experiments (37) and is close to an optimal cross-sectional configuration for a bundle of actin filaments, maximizing an individual filament’s number of next neighbors.

## Results

When filopodial growth dynamics are simulated using the approach described above, we find that, typically, an actin filament bundle undergoes several transitions between metastable configurations. Initially, a rapid growth phase is observed, during which all filaments grow together, followed by intermittent collapses of filaments inside the bundle. Hence, the bundle settles into different metastable steady states, during which the filament heights fluctuate around particular quasi-stationary values. These quasi-stationary heights are determined by a balance between the polymerization of G-actin and the combination of depolymerization of F-actin and retrograde flow (see Mean-Field Model) (19, 48). At intermediate times, large fluctuations of single filaments push the bundle out of this metastable state and one or more filaments further collapse. This collapse can be complete (a filament’s height shrinks to zero and it disappears) or partial. In the case of a partial collapse, collapsed filaments fluctuate around a new, lower height, which is largely determined by a changed supply of G-actin molecules due to the new bundle geometry. Hence, the bundle height transitions due to individual filament collapses are exclusively driven by the fluctuations of G-actin’s availability mediated by local steric constraints. This is in contrast to filament collapse due to the binding of capping proteins that instead directly halt filaments’ polymerization propensities (20). Note that the polymerization reaction is always diffusion-limited, because the filaments will simply grow until a balance is reached between the addition of new monomers and the reduction in length due to depolymerization and retrograde flow (19). Hence, our results are robust even for greater G-actin concentrations or large uncertainties in the polymerization rate parameter. We checked this with sets of simulations with *c*_0_ = 20 *μ*M, *k*^+^ = 5 *μ*M^−1^ s^−1^, and *k*^+^ = 20 *μ*M^−1^ s^−1^ (see below).

**Figure 2:**
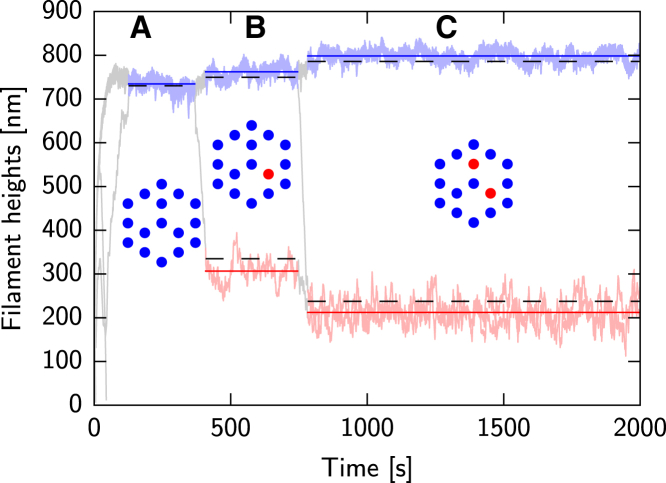
Filament heights over time from a representative simulation run with *d* = 13 nm. The upper filament trajectories at the bundle tip are highlighted in blue, the partially collapsed filament trajectories are shown in red. All filaments are accounted for in these two categories during an identified metastable state. Three distinct metastable states are visible in order of appearance in the graph: (*A*) three completely collapsed filaments with the remainder at the tip of the bundle; (*B*) one of the inner filaments collapsed partway; and (*C*) two inner filaments collapsed partway. (*Insets*) Spatial configuration of the metastable states. (*Dashed lines*) Filament heights calculated from Eq. 6. (*Shaded lines*) Transient states between metastable configurations.

Fig. 2 shows filament height dynamics from a representative simulation run. The height data clearly reveal three distinct metastable states, which were automatically identified using the DBSCAN clustering algorithm (49, 50). Uncategorized data are shown as light gray lines, while colored line plots show filament height data belonging to an identified metastable configuration. The insets show a top view of the spatial distribution of filaments inside the bundle. Blue circles indicate the lateral positions of filaments in the cluster at the filopodial top; red circles are the positions of filaments that are part of an intermediate-height cluster; and white spaces indicate the former positions of filaments that collapsed completely. Importantly, these empty spaces serve as additional channels for G-actin to diffuse inside the bundle.

After the brief initial transient, Fig. 2 shows that three filaments have completely collapsed and disappeared. The resulting bundle is found, therefore, in a highly symmetric configuration with all remaining filament tips at the top of the filopodium. Subsequently, at *t* ≈ 400 s, a large fluctuation drives a single filament tip below the stable region and it collapses toward a level of ∼300 nm. Note that at the same time, the filopodial tip (i.e., the filament cluster at the tip of the filopodium) grows slightly. At the second transition point, at *t* ≈ 750 s, a second filament collapses partway and joins the lower cluster. The two filament’s height shrinks slightly while the filopodial tip grows again. The occurrence of these metastable states, together with the transitions between them, strongly indicates the existence of multistability in this system. When filament tips inside the bundle leave the region of attraction of a stable cluster, they collapse partway and reach a different metastable state.

**Figure 3:**
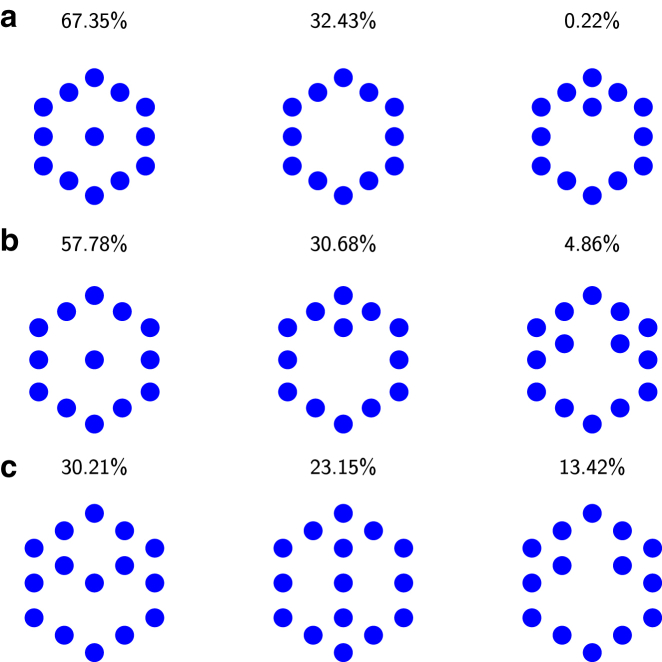
The spatial distribution of full-length filaments in the actin filament bundle inside the filopodium, displayed for the three most prevalent configurations (*a*) out of 3 found for *d* = 11 nm, (*b*) out of 7 found for *d* = 12 nm, and (*c*) out of 16 found for *d* = 13 nm. A solid circle indicates full-length filaments, and white space indicates partially or fully collapsed filaments. The numbers above the individual states indicate the percentage of total simulation time spent in that state. To see this figure in color, go online.

Over time, this leads to a hollowing out of the filament bundle, which, in turn, has interesting implications for the mechanical and geometrical properties of the filopodium. Fig. 3 shows the three most prevalent metastable configurations (considering only the filaments that are positioned at the top of the bundle), ranked by the percentage of time spent in this state throughout our study (we accounted for symmetries that make configurations equivalent) for a filament spacing of *d* = 11, 12, and 13 nm (as this covers the majority of interfilament distances observed in experiments (39)). Blue circles represent filaments that reach the top of the filopodium, while white spaces indicate completely or partially collapsed filaments (i.e., open diffusion channels inside the bundle). All of these configurations show a thinning of the filament bundle toward the filopodial top. As a general rule, when filaments in the bundle are positioned with smaller interfilament spacings with respect to each other, the inner filaments are more likely to collapse.

Because the parameter values listed in Table 1 might have large associated uncertainties or might apply only to a limited range of cell types, we tested whether our observed effects are present also for different parameter values. We systematically varied the following parameters.

### Bulk G-actin concentration

The reported G-actin concentration measured in various cell types and organisms ranges between 10 and 100 *μ*M and sometimes even higher (51, 52). However, for the purposes of our simulation study it is crucially important to distinguish not only between monomeric G-actin and polymerized F-actin, but also between G-actin that is available to polymerize and sequestered G-actin (i.e., bound to other proteins such as cofilin or thymosin-*β*4). The latter is unavailable to directly polymerize into filaments und therefore needs to be excluded. From quantitative simulation studies, Mogilner and Edelstein-Keshet (53) estimate the available unsequestered G-actin in the lamellipodium to be ≈24 *μ*M when the total available actin concentration was 250 *μ*M. From this, Zhuravlev et al. (52) estimate a valid unsequestered G-actin concentration ranging between 1 and 50 *μ*M (see Table S1 in (52)).

To check whether our results are valid for higher concentrations within this range, we ran simulations for *c*_0_ = 20 *μ*M, leading to a roughly doubled length of the filopodium in which the collapse of inner filaments together with metastable states still occur. We do not expect this to change for even higher G-actin concentrations, because inner filaments are always at a disadvantage due to the lower supply of G-actin monomers as long as bundles are able to grow when polymerization of filaments outperforms depolymerization and retrograde flow:

$$c_{0}k^{+}>k^{-}+v/\delta \approx 27.6\;{\text{s}}^{-1}.$$

For *k*^+^ = 11.6 *μ*M^−1^ s^−1^, this is the case when *c*_0_ > 2.4 *μ*M. Due to possibly large exchange rates between sequestered and unsequestered G-actin (54) the bulk G-actin concentration might be higher than assumed (and therefore the effective polymerization rate lower than measured). However, our arguments above show that our results are valid even then.

### G-actin polymerization rate

We ran additional simulations with a polymerization rate of *k*^+^ = 5 *μ*M^−1^ s^−1^ and *k*^+^ = 20 *μ*M^−1^ s^−1^. In the latter case, the length of the filopodium is enhanced, but the reported effects due to steric interactions are still observable. The filopodia become very short (<300 nm) in the former case, and the probability of partially collapsed filaments recovering (instead of collapsing completely) to the filopodial tip becomes significantly higher, which is to be expected because small positive height fluctuations are then sufficient to reach the upper stable configuration. We also ran simulations with a doubled G-actin concentration *c*_0_ = 20 *μ*M and an approximately halved G-actin polymerization rate of *k*^+^ = 5 *μ*M^−1^ s^−1^, yielding filopodia of a length of ∼1.4 *μ*m with hollow bundles.

### Diffusion constant

To check the influence of the diffusion constant, we ran simulations with *D* = 2.5 × 10^6^ nm^2^ s^−1^. This also led to a significantly reduced filament height (which is to be expected), similar to the reduction in the polymerization rate.

Therefore, we conclude that our observed actin bundle remodeling effects are indeed robust against relatively large changes in the G-actin parameter values.

### Changes in mechanical stability

In an earlier work, Mogilner and Rubinstein (11) analyzed the mechanical stability of a filopodial bundle. At the maximum stable length, *L*, the force enacted on the bundle via the membrane *F* is equal to the buckling force of the bundle (55)

(2) $$F=\frac{\pi^{2}B_{s}}{4L^{2}},$$

where $B_{s}=EI$ is the bending stiffness, *E* is the Young’s modulus, and *I* is the second moment of area (interactions with filament cross-linking molecules are not considered). The resulting buckling length is $L=\pi \sqrt{B_{s}/4F}$. When considering single actin filaments in the bundle as filled rods, the bending force and hence the buckling length of different lateral arrangements of filaments differ only in their second moment of area. A single filament with radius *r*_*F*_, cross-sectional area *A*, and distance $\vec{a}$ from the bundle center contributes to the bundle’s second moment of area via the Huygens-Steiner theorem

$$I_{s}\left(a\right)=\int_{A}{\left|\vec{r}-\vec{a}\right|}^{2}dA=\frac{\pi }{4}r_{F}^{4}+\pi r_{F}^{2}a^{2}.$$

Hence, a full bundle in a hexagonal arrangement with interfilament spacing *d* with 19 filaments has a second moment of area of

$$\begin{array}{c} I_{F}=I_{s}\left(0\right)+6I_{s}\left(d\right)+6I_{s}\left(2d\right)+6I_{s}\left(\sqrt{3}d\right)\\ =\pi r_{F}^{2}\left(\frac{19}{4}r_{F}^{2}+48d^{2}\right). \end{array}$$

A hollow bundle in a hexagonal arrangement with all seven inner filaments collapsed exhibits a second moment of area of

$$\begin{array}{c} I_{O}=6I_{s}\left(2d\right)+6I_{s}\left(\sqrt{3}d\right)\\ =\pi r_{F}^{2}\left(3r_{F}^{2}+42d^{2}\right). \end{array}$$

Hence, the buckling length ratio between hollow and intact filopodial bundles is (assuming *d* = 13 nm)

$$g=\sqrt{\frac{3r_{F}^{2}+42d^{2}}{\frac{19}{4}r_{F}^{2}+48d^{2}}}\approx 0.93.$$

Therefore, the maximum stable length of a totally hollowed-out bundle is reduced to 93% of the intact bundle, i.e., the effect on the mechanical stability is rather weak. If, on the other hand, the bundle is laterally coalesced into a hexagonal arrangement by, for example, strong cross-linker interactions or molecular motors, the second moment of area becomes

$$\begin{array}{c} I_{C}=I_{s}\left(0\right)+6I_{s}\left(d\right)+5I_{s}\left(\sqrt{3}d\right)\\ =\pi r_{F}^{2}\left(3r_{F}^{2}+21d^{2}\right), \end{array}$$

and the above-mentioned length ratio becomes *g* ≈ 0.66, producing a much larger effect because of the reduced cross-sectional area of the filament bundle.

### Mean-field model

**Figure 4:**
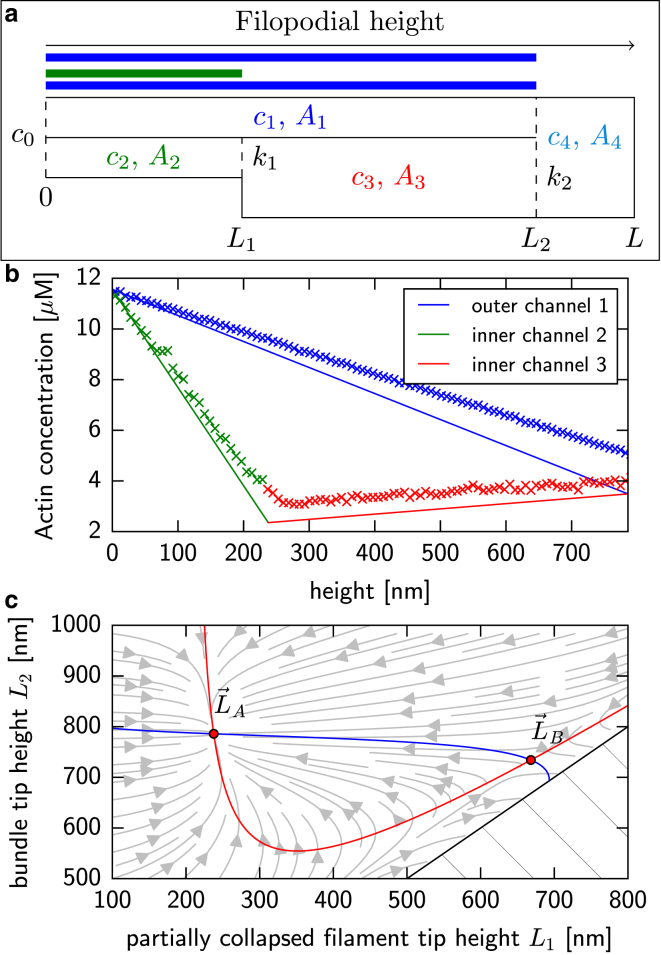
(*a*) Sketch of the one-dimensional diffusion channels considered as part of the mean-field model for a filopodium with two distinct filament heights. The two channels exhibit *x*-dependent piecewise linear concentrations *c*_1_, *c*_2_, *c*_3_, and *c*_4_. The lower channel changes its cross section at position *L*_1_ due to the presence of one or more filament tips. At position *L*_2_, both channels merge and the cross-sectional area changes again with *A*_1_ + *A*_3_ < *A*_4_. The membrane is at *L* = *L*_2_ + 25 nm. Polymerization of G-actin is implemented via sinks with strength *k*_1_ and *k*_2_ at positions *L*_1_ and *L*_2_, respectively. (*b*) Mean-field G-actin concentration profiles in the three channels for metastable configuration (C) displayed in Fig. 2 together with data from stochastic simulations. (*c*) Plot of the roots of the two stability conditions (Eq. 6) as a function of *L*_1_ and *L*_2_ for the same configuration. The points of filament stability where both conditions are true simultaneously are indicated by red dots. Light gray lines show the flow of the stability conditions. The hatched area indicates the unphysical regime *L*_1_ ≥ *L*_2_.

To provide deeper understanding into the processes that lead to filament collapse inside the bundle’s core, we next introduce the following simplified model. We assume that G-actin diffusion occurs fast, such that linear concentration profiles are quickly established along the filopodia axis. The arrangement of filaments is such that G-actin cannot pass between two neighboring filaments. Fig. 4 *a* shows a sketch of this channel configuration. Hence, there exist at least two separate channels for diffusion in the filopodium: G-actin can diffuse along and outside of the filament bundle, as well as via any inner space that opens up due to filament collapse. This inner channel can then host filaments that are partially collapsed, which yields a changing cross-sectional area of the diffusion channel. The outer channel exhibits a cross-sectional area *A*_1_. The area of the inner lower channel is given by *A*_2_, which widens to *A*_3_ at the partially collapsed filaments tip position $L_{1}$. The two channels merge to a single diffusion channel with area $A_{4}=\pi r_{F}^{2}$ at the bundle’s tip $L_{2}$. In a metastable state, the filament tips in the partially collapsed level as well as the bundle’s top both consume just enough G-actin to hold their height stable. The G-actin concentrations in the corresponding channels $c_{1}$, $c_{2}$, $c_{3}$, and $c_{4}$ then follow the set of diffusion equations

(3) $$\begin{array}{cc} \frac{d^{2}c_{1}}{dx^{2}}=0,\;x\in \left[0,L_{2}\right] & \frac{d^{2}c_{2}}{dx^{2}}=0,\;x\in \left[0,L_{1}\right]\\ \frac{d^{2}c_{3}}{dx^{2}}=0,\;x\in \left[L_{1},L_{2}\right] & \frac{d^{2}c_{4}}{dx^{2}}=0,\;x\in \left[L_{2},L\right], \end{array}$$

with the conditions at the boundaries of the different channels

(4) $$\begin{array}{l} c_{1}\left(0\right)=c_{2}\left(0\right)=c_{0},\\ c_{2}\left(L_{1}\right)=c_{3}\left(L_{1}\right),\\ c_{1}\left(L_{2}\right)=c_{3}\left(L_{2}\right)=c_{4}\left(L_{2}\right), \end{array}$$

and the flux conditions across these boundaries

(5) $$\begin{array}{l} A_{2}\frac{dc_{2}}{dx}\left(L_{1}\right)-A_{3}\frac{dc_{3}}{dx}\left(L_{1}\right)=-\frac{k_{1}}{D}c_{3}\left(L_{1}\right)\;,\\ A_{1}\frac{dc_{1}}{dx}\left(L_{2}\right)+A_{3}\frac{dc_{3}}{dx}\left(L_{2}\right)=-\frac{k_{2}}{D}c_{4}\left(L_{2}\right)\;,\\ \frac{dc_{4}}{dx}\left(L\right)=0\;. \end{array}$$

The coefficient of the reactive boundary condition describing the removal of G-actin by the *N*_1_ partially collapsed filament tips is given by $k_{1}=N_{1}k^{+}/N_{A}$, where *N*_*A*_ is Avogadro’s constant. Similarly, the removal of molecules via the *N*_2_ filaments at the bundle tip is implemented by a reactive boundary condition at $L_{2}$ with the coefficient $k_{2}=f_{m}\left(N_{2}\right)N_{2}k^{+}/N_{A}$. The factor $f_{m}\left(N_{2}\right)$ stems from the steric interactions of G-actin with the membrane at the top of the filopodium, and is calculated via

$$f_{m}\left(N_{2}\right)=0.550+2.524\times 10^{-2}N_{2}-5.131\times 10^{-4}N_{2}^{2},$$

where the coefficients result from a fit of a polynomial of order 2 to data generated in simulations and listed in Table 2. Equations 3–5 can be written as a system of linear equations for the concentrations at the filament tips $c_{3}\left(L_{1}\right)$ and $c_{1}\left(L_{2}\right)$.

Fig. 4 *b* shows the resulting linear concentration profiles in the respective channels for the example configuration (C) shown in Fig. 2, together with data obtained from our simulations. As discussed above, filament heights are (meta)stable when the polymerization of G-actin with rate *k*^+^ is counterbalanced by the effective reduction in length due to the depolymerization reaction with rate *k*^−^ and retrograde flow with speed *v*. Thus, the stability criteria are given by

(6) $$\begin{array}{c} f_{m}\;k^{+}c_{1}\left(L_{2}\right)\frac{A_{1}}{A_{4}}=k^{-}+\frac{v}{\delta },\\ k^{+}c_{3}\left(L_{1}\right)=k^{-}+\frac{v}{\delta }, \end{array}$$

where $c_{1}\left(L_{2}\right)$ and $c_{3}\left(L_{1}\right)$ are the concentrations of G-actin at the filament tip positions $L_{2}$ and $L_{1}$ in channels 1 and 3, respectively. The area ratio is necessary due to the geometry of the bundle. The effective reduction of the polymerization rate at the $L_{2}$ tip position due to membrane interaction is taken into account by the phenomenological factor $f_{m}$. These criteria together with the solution from Eqs. 4–6 describe two nonlinear curves in the $0<L_{1}<L_{2}$ sector of the plane spanned by $L_{1}$ and $L_{2}$. If there exists a stable configuration, the two curves intersect in two points, which are given by ${\vec{L}}_{A}=\left(L_{1,A},L_{2,A}\right)$ and ${\vec{L}}_{B}=\left(L_{1,B},L_{2,B}\right)$. The point ${\vec{L}}_{A}$ is a stable configuration within the limits of this model, while the point ${\vec{L}}_{B}$ is a saddle point that mediates the collapse of filaments from the bundle tip. Fig. 4 *c* shows the two curves and their intersection points for configuration (C) shown in Fig. 2. The light gray arrows indicate the vector field

$$\left(\frac{f_{m}k^{+}c_{1}\left(L_{2}\right)}{k^{-}+v/\delta }\frac{A_{1}}{A_{4}}-1,\;\frac{k^{+}c_{3}\left(L_{1}\right)}{k^{-}+v/\delta }-1\right),$$

which corresponds to the flow of the filament stability conditions (6). In the stochastic simulation model, the point ${\vec{L}}_{B}$ becomes quasi-stable. As soon as a fluctuation in the height of a single filament causes it to fall ∼100 nm below $L_{1,B}$, the single filament will collapse to the height $L_{1,A}$ and the bundle tip will move to a length $L_{2,A}$.

The black dashed lines in Fig. 2 show the predicted filament heights for three different metastable configurations, with very good agreement with our simulation data. The observed overestimation of the height of the partially collapsed filaments and the underestimation of the height of the bundle tip stems from the inability of the theory to capture the more complicated spatial structure inside a bundle. This is also the reason for the discrepancy between the mean-field concentrations and the data from stochastic simulations in Fig. 4 *b*.

To find the parameter regimes in which metastable bundle configurations exist (rather than inner filaments simply collapsing completely), we extracted the mean-field stability boundary of the bundle configuration (C) in Fig. 2 via finding the value of $k^{+}\left(c_{0}\right)$ at which expressions in Eq. 6 start to have solutions. It is well described by the curve given as $c_{0}k^{+}\approx 63.8\;{\text{s}}^{-1}$. However, this only applies to this particular bundle configuration. When we repeat the same analysis over all bundle configurations, we observe in simulations with *D* = 5 × 10^6^ nm^2^ s^−1^ and *d* = 13 nm but varying *c*_0_ and *k*^+^, the minimum value is $\alpha =c_{0}k^{+}\approx 46.9\;{\text{s}}^{-1}$. This value of *α* can be viewed as the upper limit for the phase boundary between immediate collapse of inner filaments $\left(c_{0}k^{+}<\alpha \right)$ and the existence of metastable filament heights $\left(c_{0}k^{+}>\alpha \right)$.

## Discussion

Our results show that the sterically hindered movement of free G-actin molecules leads to intriguing effects: when G-actin cannot pass between filaments in the filopodial shaft bundle, the polymerization of barbed ends of F-actin inside the bundle may not be sufficient to counteract retrograde flow. However, instead of the complete collapse of all filaments, novel metastable intermediate-height states emerge. Interior filaments that arise from an initial transient growth regime, initially have similar heights compared with the stable exterior filaments. However, height fluctuations will eventually drive single or multiple interior filaments below a critical stable height $L_{1,B}$, after which they collapse to a new metastable height $L_{1,A}$. We verified that this effect is present for different values of the interfilament spacing *d* observed in experimental measurements of actin bundles. Our mean-field model enables us to approximately calculate the expected critical heights for a given configuration. As these new states are only metastable, the partially collapsed filaments will eventually collapse fully and disappear. This, in turn, opens up new diffusion channels for G-actin molecules, raising their concentration in the bundle interior and thereby enhancing the stability of any remaining interior filaments. Due to the raised supply of G-actin, the average height of the topmost filament tips is raised as well.

The configuration of the filament bundle at any given time depends on the history of all prior filament collapses starting from the initial transient. Therefore, the ensemble of observed bundle configurations is highly diverse, with a small selection of configurations shown in Fig. 3. Over time, the interior of the bundle becomes hollowed-out with some interior filaments collapsing partially or completely. Thus, volume exclusion and the resulting change in the free diffusion of G-actin facilitate sculpting of the actin bundle inside filopodia. This reaction-diffusion sculpting mechanism adds complexity to the formin/capping protein-mediated filament dynamics investigated in Zhuravlev and Papoian (20).

Furthermore, we should consider the possibility that internal forces due to filament cross-linking proteins or molecular motors may laterally constrict bundles with collapsed internal filaments. Hence, as the filopodium ages, two outcomes are likely: (1) after the bundle becomes hollowed-out due to reaction-diffusion sculpting, it continues to remain hollow (for example, because of weak cross-linking activity), experiencing only a small reduction of its initial mechanical stability, as discussed above. (2) The bundle shrinks radially due to inward internal forces collapsing the hollow cavity, which, in turn, would lead to a strong reduction of the bundle’s mechanical stability. This second scenario would imply an overall conical shape of aged filopodial bundles, with a thicker portion position near the filopodial base and a thinner section positioned at the filopodial tips, hence, explaining the corresponding common observations of conically shaped actin filament bundles in superresolution and fluorescence microscopy imaging of filopodia; see, e.g., Fig. 1 *A* in Suraneni et al. (56) or Fig. 1 *C* in Svitkina et al. (57). Tapering of long filopodial protrusions has also been reported in sea urchins (58), epithelial cells (59), and plant cells (60).

## Author Contributions

U.D. wrote and performed the simulations; U.D. and R.E. evaluated the computed results; and U.D., G.A.P., and R.E. designed the project and cowrote the article.

## Figures and Tables

**Table 1:** Parameter Values Used Throughout This Study

Description	Value	Reference
G-actin parameters		
Diffusion constant	*D* = 5 × 10^6^ nm^2^ s^−1^	(11)
Bulk G-actin	*c*_0_ = 10 *μ*M	(51)
Geometry		
Filopodial radius	*R* = 75 nm	(9)
Filament spacing	*d* = 11, 12, 13 nm	(37, 38, 39)
Filament radius	*r*_*F*_ = 3.5 nm	(34, 35, 36)
Filament number	*N* = 19	(10)
Filament dynamics		
Binding radius	ϱ = 2*r*_*F*_	
Actin on-rate	*k*^+^ = 11.6 *μ*M^−1^ s^−1^	(61, 62)
Actin off-rate	*k*^−^ = 1.4 s^−1^	(61, 62)
Retrograde flow	*v* = 70 nm s^−1^	(19)
Polymer length	*δ* = 2.7 nm	(19)
Membrane interaction		
Membrane force	*f* = 10 pN	(19, 44)
Fluctuation size	*σ* = 20 nm	(19, 44)
Temperature	*k*_B_*T* = 4.1 pN nm	(11)

The numbers in the third column indicate references for the cited parameter values.

**Table 2:** Membrane Factor Values Obtained from Simulations

*N*_2_	*f*_*M*_(*N*_2_)	*N*_2_	*f*_*M*_(*N*_2_)
12	0.779	16	0.823
13	0.792	17	0.831
14	0.804	18	0.838
15	0.814	19	0.845
